# Immune Parameters That Distinguish Multiple Sclerosis Patients from Patients with Other Neurological Disorders at Presentation

**DOI:** 10.1371/journal.pone.0135434

**Published:** 2015-08-28

**Authors:** Athanasia Mouzaki, Maria Rodi, Nikolaos Dimisianos, Andreas Emmanuil, Dimitra Kalavrizioti, Rosa Lagoudaki, Nikolaos C. Grigoriadis, Panagiotis Papathanasopoulos

**Affiliations:** 1 Division of Hematology, Department of Internal Medicine, Medical School, University of Patras, Patras, Greece; 2 Department of Neurology, Patras University Hospital, Patras, Greece; 3 Laboratory of Hematology, Patras University Hospital, Patras, Greece; 4 Department of Neurology, AHEPA University Hospital, Thessaloniki, Greece; LMU Munich, GERMANY

## Abstract

**Background/Aim:**

Multiple sclerosis (MS) is an inflammatory, demyelinating disease of the central nervous system. Effector T helper cells, mainly Th1 and Th17, cytotoxic T-cells, B-cells, macrophages, microglia, and the cytokines they secrete, are implicated in the initiation and maintenance of a deregulated immune response to myelin antigens and the ensuing immune-mediated demyelination. In this study, we investigated whether signature cytokines exist in MS patients at presentation to gain an insight into the underlying immunopathogenic processes at the early stage of the disease.

**Methods:**

We collected serum and cerebrospinal fluid (CSF) samples from 123 patients at presentation, eventually diagnosed with MS or non-inflammatory (NIND) or inflammatory neurological diseases (IND) or symptomatic controls (SC). The levels of cytokines IFN-γ, TNF-α, TGF-β1, IL-2, IL-4, IL-6, IL-10 and IL-17 were measured, and cytokine ratios, such as Th1/Th2, Th1/Th17, and Type-1/Type-2, were calculated. All parameters were tested for their correlations with the intrathecal IgG synthesis.

**Results:**

Cytokine levels in CSF were lower than in serum in all the patients, with the exception of IL-6. Serum or CSF cytokine levels of MS patients did not differ significantly from NIND or SC, with the exception of serum IFN-γ and TNF-α that were significantly higher in NIND. IND patients presented with the highest levels of all cytokines in serum and CSF, with the exception of serum IL-10 and CSF IL-17. MS patients had a significantly lower serum Th1/Th2 ratio compared to the NIND and IND groups, and significantly lower serum Type-1/Type-2, IFN-γ/IL-10 and CSF Th1/Th17 ratios compared to IND patients. MS patients had a significantly higher CSF IL-17/IL-10 ratio compared to IND patients. The IgG index was higher in MS patients compared to the control groups; the differences reached statistical significance between the MS and the NIND and SC groups. Reiber-Felgenhauer analysis of the QIgG and QAlb indices revealed higher intrathecal IgG synthesis in MS patients, and higher blood-CSF barrier dysfunction in IND patients. The IgG index correlated with CSF IL-4 in MS patients only.

**Conclusions:**

We found no signature cytokines or profiles thereof in MS patients at presentation. Only IND patients presented with a clear Th1 cytokine polarization in serum and CSF. The parameters that distinguished MS patients from patients with other neurological disorders were IgG intrathecal synthesis, the IgG index and its correlation with CSF IL-4 levels.

## Background

MS is a chronic, inflammatory, demyelinating disease of the CNS, affecting predominantly young adults with a female preponderance [1]. The actual cause of MS is unknown, but it has been established that genetic predisposition and environmental factors play a crucial role, in conjunction with a failure of immune tolerance mechanisms to suppress and effectively abolish the self-reactive cells [2].

Autoreactive Th cells are considered responsible for the initiation and maintenance of autoreactivity to CNS myelin, with the involvement of a variety of other immune cells, including macrophages, B-cells, NK cells, cytotoxic T-cells and microglial cells [3]. MS was originally thought to be a Th1-driven disease, a notion supported primarily by observations from an animal model of MS, experimental allergic/autoimmune encephalomyelitis (EAE) [4,5]. This was later challenged in the EAE model, when it was shown that IL-12 knockout mice (unable to generate Th1 cells) were still susceptible to EAE, whereas IL-23 knockout mice were not [6]. This led to the discovery of a new subset of Th cells, Th17, that secrete IL-17, IL-6 and TNF-α [7,8].

In human MS patients, Th17 cells and also IFN-γ and IL-17 co-expressing Th cells have been detected in lesions, especially active ones [9, 10]. In addition, peripheral blood T-cells isolated from MS patients with active disease, when cultured without external stimulation, secrete high amounts of IFN-γ and TNF-α [11] but low amounts of IL-17 [12]. Upon non-specific (anti-CD3 antibodies or mitogens) or specific stimulation (with cognate antigens) peripheral blood T-cells show a strong Th1 or Th17 polarization or IFN-γ and IL-17 co-expression [10–13].

Cytokines are secreted signaling proteins that regulate immune responses and inflammatory reactions, and are characterized by the cells that produce them and also by the effects they confer in a given setting, promoting or suppressing immunological reactions [8,14]. In this context, cytokines have been characterized either as pro-inflammatory (e.g. IFN-γ, the signature cytokine of Th1 cells, IL-17, the signature cytokine of Th17 cells, TNF-α, IL-6) or as anti-inflammatory (e.g. IL-4, the signature cytokine of Th2 cells, IL-10 and TGF-β, signature cytokines of T regulatory cells). Cytokines have often dual and opposite actions, the most characteristic example being IL-2, a pleiotropic cytokine that, in humans, is secreted by naive Th cells when activated, stimulates proliferation and effector functions of Th, cytotoxic T-cells, B-cells and NK cells, promotes activation-induced cell death, but it also suppresses Th17 differentiation and is an essential growth factor of regulatory T cells [15,16]. In addition, antigen presenting cells (APCs) and the antigen itself govern the subsequent effector phase of the immune reaction by creating a particular cytokine milieu [17]. It would, therefore, be reasonable to assume that different pathological processes (infectious, autoimmune, structural, etc.) affecting the nervous system are characterized by distinct cytokine profiles that reflect the underlying immunopathogenic processes at different stages of the disease.

The aim of this study was to assess whether there is a distinct cytokine profile in MS patients in the early stage of the disease that distinguishes MS patients from patients suffering from other inflammatory and non-inflammatory neurological disorders. To this end, we measured serum and CSF Th-type cytokine levels in samples collected from patients at presentation that were eventually diagnosed with MS or other non-inflammatory or inflammatory neurological diseases or symptomatic controls, and analyzed our results for correlations with intrathecal IgG synthesis indices.

## Methods

### Ethics Statement

All subjects gave written informed consent before enrollment in the study. The study protocol was approved by the Patras University Hospital (PUH) Ethics (Re: 296/23.9.08) and Scientific (Re: 451/17.10.08) Committees as part of a general application to collect biological samples from patients attending the Neurology Clinic to study in vitro the role of T helper cell populations and cytokines in the pathogenesis, prognosis and natural course of multiple sclerosis. The Hospital abides by the Helsinki declaration on ethical principles for medical research involving human subjects.

### Study subjects

Forty six patients (27F/19M), who were eventually diagnosed as having relapsing-remitting MS (RR-MS), according to the 2005 revised McDonald criteria [18], were included in the study. The patients were all newly diagnosed after been admitted to the Neurology Department of PUH for evaluation of symptoms suggestive of MS. They all underwent extensive tests that included brain and spinal cord MRI, lumbar puncture (LP) and CSF analysis for estimation of the IgG index and detection of oligoclonal bands (OCBs), visual evoked potentials, and appropriate tests to exclude other diseases that could better account for their symptoms (e.g. systemic lupus erythematosus, sarcoidosis, vasculitis, viral infections, vitamin B_12_ deficiency, etc.). Exclusion criteria included diagnoses other than MS, active infection or inflammation of any kind, and current or recent treatment with immunosuppressive or immunomodulatory drugs. A detailed history was taken to record information about other symptoms in the past suggestive of MS, the family history and other illnesses, and a thorough neurological examination was performed to determine the patients’ level of disability according to the Expanded Disability Status Scale (EDSS) [19] (Table 1). The MS patients included in the study were selected among those with recent disease onset, i.e. those with symptom onset of no longer than 2 months.

**Table 1:** Demographic and disease characteristics of MS patients and control groups.

Subjects		N (F/M)	Age (years)	EDSS score	OCB^a^	CSF cell count^b^
MS*		46 (27/19)	34.04 (10.18)	2.5 (1–5.5)	76%	9.5 (8.33)6 (0–29)
Controls		77 (43/34)	38.44 (14.56)	NA	-	
NIND		40 (23/17)	37.8 (14.37)	NA	-	1.64 (1.5)1 (0–5)
Diagnosis	Ischemic disease (lacunar, embolic infarcts)	15				
	Migraine	12				
	Idiopathic intracranial hypertension	3				
	Peripheral neuropathy	3				
	Epilepsy (idiopathic)	2				
	Chorea	1				
	Pontine cyst	1				
	Brain tumor	1				
	Idiopathic tremor	1				
	Spastic paraplegia	1				
IND		22 (12/10)	44.18 (15.63)	NA	-	50.5 (71)13.5 (1–210)
Diagnosis	Viral encephalitis^**+**^	6				
	Viral myelitis^**+**^	5				
	Multiple radiculoneuritis^**§**^	3				
	Guillain-Barré syndrome	3				
	Neurosyphilis	2				
	CIDP^#^	1				
	Limbic encephalitis	1				
	CNS vasculitis	1				
SC		15 (8/7)	33.54 (10.51)	NA	-	1.46 (1.39) 1 (0–4)

Values for age are given as mean (SD) and for EDSS as median (range). N, sample size; F, female; M, male; MS, multiple sclerosis; NIND, non-inflammatory neurological diseases; IND, inflammatory neurological diseases; SC, symptomatic controls; NA, not applicable.

*Relapsing-remitting MS (RR-MS).

^**+**^The diagnosis of viral infection was based on the patients’ history and clinical presentation, CBC, amount and type of cells in the CSF, MRI findings, and course of the disease. The patients were treated with acyclovir iv (Zovirax) at 10 mg/Kg/8h for a total of 14 days. All had a favorable outcome. Laboratory tests excluded the presence of bacteria or fungi. The patients’ sera were screened for antibodies to HSV, EBV, CMV, VZV, HTLV I/II, HIV I/II, Borrelia, Toxoplasma, TB, HBV, HCV: They tested negative for IgM antibodies and positive for IgG antibodies to HSV, EBV, CMV, VZV, Toxoplasma and HBV. PCR in CSF was performed for HCV, HBV, HIV I/II, HSV I/II and TB (negative).

^#^CIDP = Chronic inflammatory demyelinating polyradiculoneuropathy. The patient received a 5-day treatment with IVIG at a total dose of 2g/kg.

^**§**^Patients with multiple radiculoneuropathies were given a 3-day iv methylprednisolone regimen of 1g/day followed by oral tapering.

^a^Percentage of patients with >2 CSF-restricted IgG bands on isoelectric focusing.

^b^Mononuclear cells/ml CSF. Values are given as mean (SD) and median (range).

The control group consisted of 77 subjects (43F/34M). They also underwent neuroimaging studies, LP and all other appropriate tests to make a diagnosis. These control subjects were assigned to 3 separate groups according to their diagnosis (Table 1). The first group (n = 40) included patients who had a non-inflammatory neurological disease (NIND), the second group (n = 22) consisted of patients with an inflammatory neurological disease (IND) other than MS, and the third group (n = 15) consisted of symptomatic controls (SC), according to the consensus definitions of the BioMS-eu network [20]. In the SC group we included patients with no inflammatory, structural or other abnormality of the CNS. Symptoms of SC included vertigo, tension headache, numbness, visual disturbances, depression, stress; however no clinical signs or evidence from imaging or laboratory studies indicated a pathological process. Patients with depression were subsequently referred to and followed up by the Psychiatric dpt. of PUH where it was confirmed that they suffered from mild or moderate Major Depressive Disorder.

### Determination of cytokine levels

All serum and CSF samples from patients and controls were collected under identical conditions between 10–11 in the morning and stored aliquoted at -75°C until processing. For measurement of the cytokine concentrations each aliquot was thawed and used only once. Determination of the cytokines IFN-γ, TNF-α, IL-2, IL-4, IL-6, IL-10 and IL-17A serum and CSF levels was performed on a BD FACSArray Bioanalyzer using the Cytometric Bead Array (CBA) assay (human Th1/Th2/Th17 Cytokine Kit, BD Biosciences, San Diego, CA, USA). TGF-β1 serum and CSF levels were measured by ELISA (R&D Systems Quantikine TM, Minneapolis, MN, USA). Cytokine ratios were also calculated (Table 2). All measurements were done in 3plicate and the intra-assay coefficients of variation were <5% for all samples. For the CBA method, data analysis was performed using the FlowJo V7.5 software (Tree Star Inc., Ashland, OR, USA) to determine the mean fluorescence intensity (MFI) of each cytokine/dilution point of the recombinant cytokine control (standard), and of each cytokine/sample. The MFI vs. the concentrations of the standards (in pg/ml) were plotted using the curve fitting software CurveExpert 1.40 to generate the standard curves from which the concentration of each cytokine/sample/MFI value was calculated. Two standard curves were created for each cytokine, one for serum and another for the CSF measurements, because the CBA protocol recommended the use of a different buffer (Serum Enhancement Buffer) at the stage of beads preparation for serum samples. Our standard curves are shown in S1–S7 Figs. The detection limits of the cytokines were assayed from the standard curves, and were: <1pg/ml for IFN-γ, TNF-α, IL-2, IL-4, IL-6 and IL-10 (in serum or CSF), 20 pg/ml for IL-17A in serum and <1 pg/ml in CSF. The detection limit of TGF-β1 that was measured by ELISA is given by the manufacturer as 4.61 pg/ml for serum or CSF.

**Table 2:** Cytokines and cytokine ratios measured in patients and controls.

Measurements (in serum and CSF)	
Cytokines	IFN-γ, TNF-α, TGF-β1, IL-2, IL-4, IL-6, IL-10, IL-17A
Th1/Th2 ratio	[IFN-γ+TNF-α]/IL-4
Th1/Th17 ratio	[IFN-γ+TNF-α]/IL-17A
Th17/Th2 ratio	IL-17A/IL-4
Type 1/Type 2 ratio	[IL-2+IFN-γ+TNF-α+IL-17A+IL-6]/[IL-4+IL-10+logTGF-β1]
More ratios	IFN-γ/IL-10, IL-17A/IL-10

### Statistical analysis

Data were analyzed using the GraphPad Prism v. 5.03 (San Diego, CA, USA). X^2^ (chi-square) analysis was applied to estimate the differences in sex among the 4 groups. The ages, disease activity/time since onset of symptoms, cytokine levels in serum and CSF, and the various cytokine ratios between the 4 groups were compared using the Kruskal-Wallis non-parametric test. When the null-hypothesis of the Kruskal-Wallis test was rejected, post-hoc pairwise comparisons were conducted by applying the Mann-Whitney U test after controlling for type I error using the Holm’s sequential Bonferroni method. Spearman’s r correlation coefficients were calculated to assess associations between variables. The statistical significance level was set at α = 0.05.

## Results

### Differences between the patient groups with regard to age, sex, or disease activity/time since onset of symptoms

There were no statistically significant differences between the ages of the MS, NIND and IND groups; the ages of the SC group were significantly lower than the ages of the IND group, whereas they did not differ significantly from the ages of the MS and NIND groups. Analysis between the patient groups with regard to sex and disease activity/time since onset of symptoms did not reveal any significant statistical differences.

### Serum and CSF cytokines

**Fig 1:**
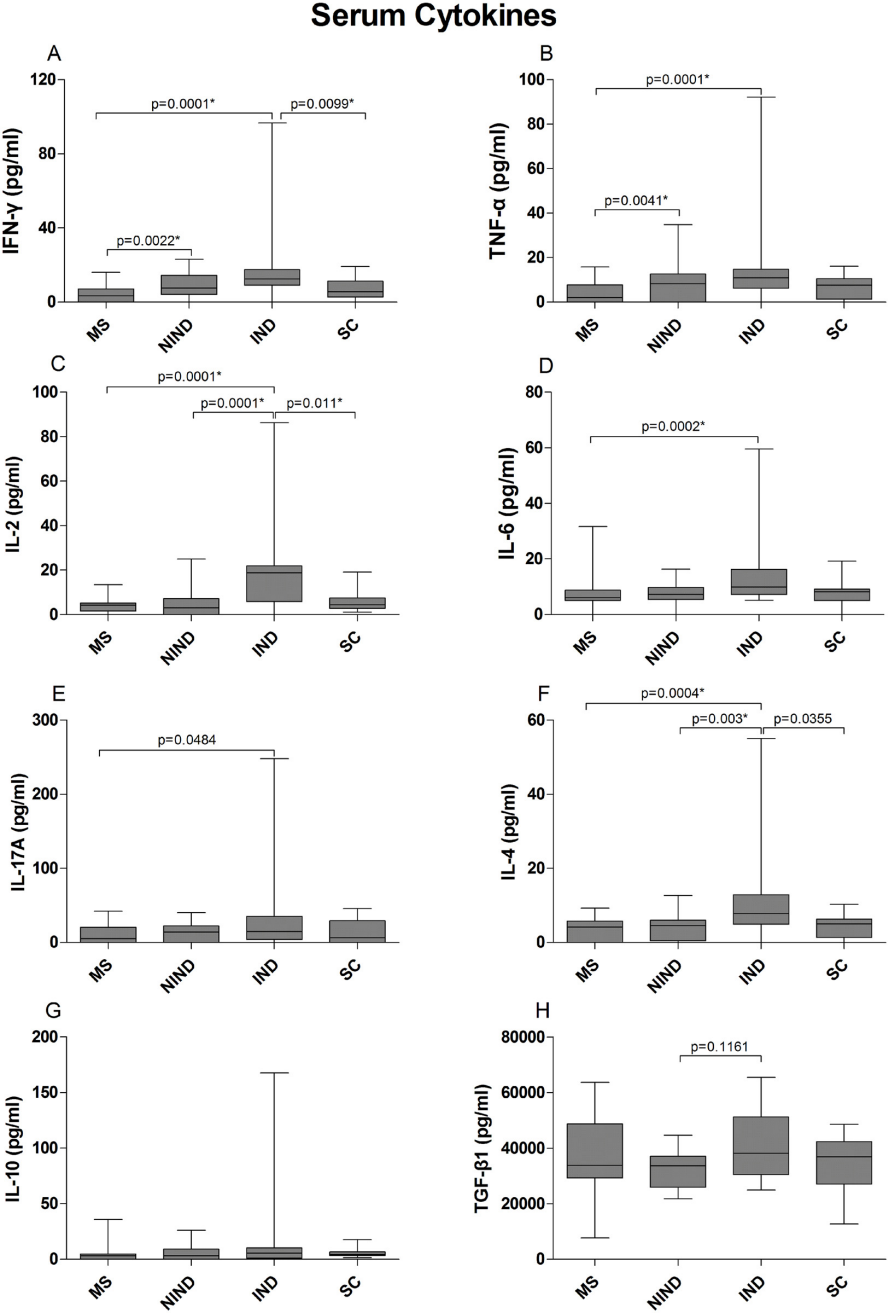
Cytokine concentrations (pg/ml) in sera of MS patients and control groups. The data are presented as box plots with whiskers, showing the median with the upper (75) and lower (25) percentiles. The uncorrected p values are shown where statistical significance was reached. The asterisk (*) denotes statistical significance retained after controlling for type I error.

**Fig 2:**
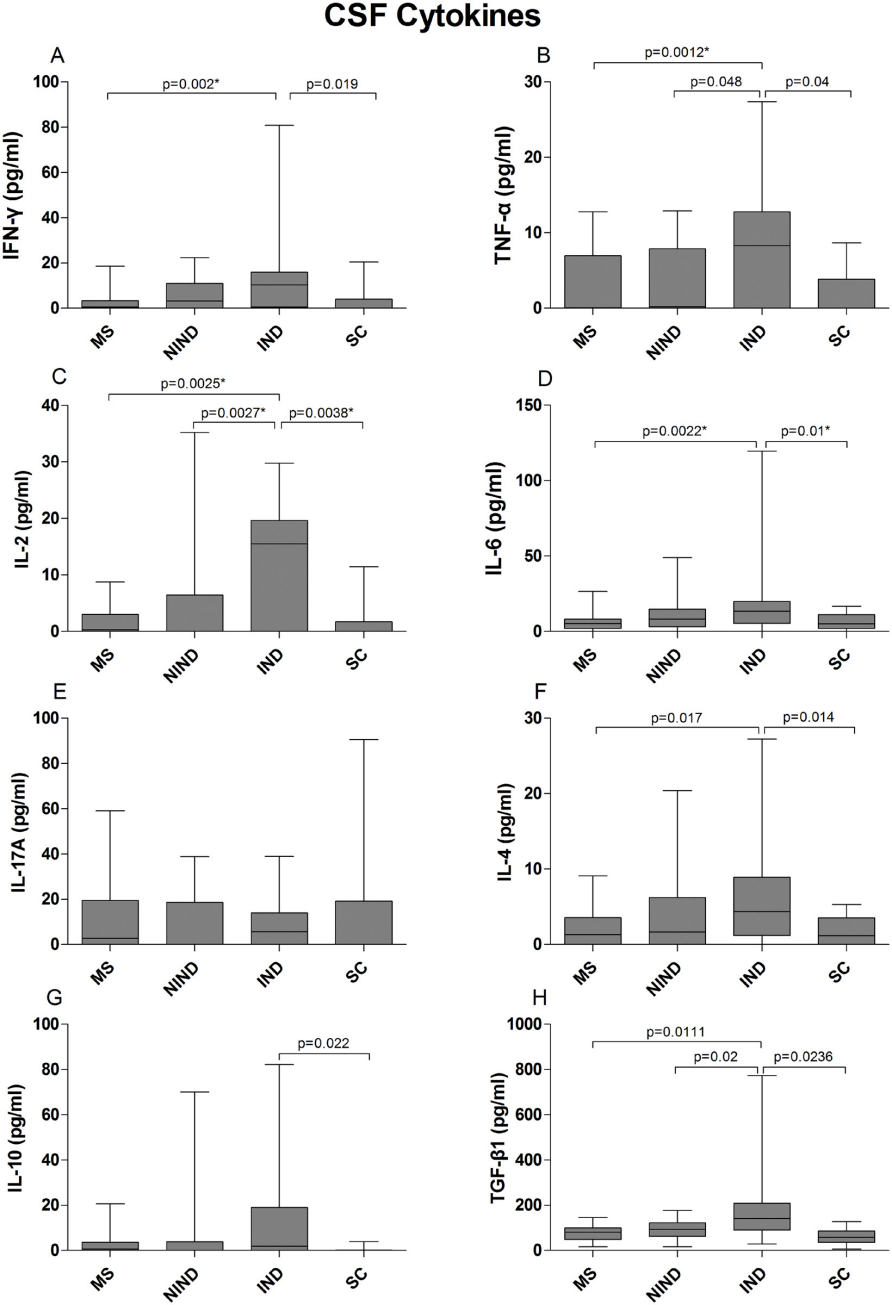
Cytokine concentrations (pg/ml) in CSF of MS patients and control groups. The data are presented as box plots with whiskers, showing the median with the upper (75) and lower (25) percentiles. The uncorrected p values are shown where statistical significance was reached. The asterisk (*) denotes statistical significance retained after controlling for type I error.

In all patient groups, the levels of CSF cytokines were significantly lower than serum cytokines, with the exception of CSF IL-6 levels (Figs 1 and 2 and S1 Table). MS patients had the lowest concentrations of serum cytokines with the exception of TGF-β1; MS serum cytokine levels were similar to NIND and SC levels, with the exception of IFN-γ and TNF-α that were significantly higher in NIND patients (Fig 1 and S1 Table). The highest concentrations of cytokines in the serum were observed in the IND group. The differences reached statistical significance (retained after controlling for type I error) for IL-2 and IL-4 compared to MS, NIND and SC groups, for TNF-α compared to MS patients, for IFN-γ compared to MS and SC patients, and for IL-6 compared to MS patients. Serum concentrations of IL-10 were also higher in the IND group, but the differences were not statistically significant (Fig 1).

MS patients had low concentrations of CSF cytokines also, at levels similar to NIND and SC groups (Fig 2 and S1 Table). IND patients had the highest concentrations of CSF cytokines. The differences reached statistical significance (retained after controlling for type I error) for IL-2 compared to all other groups, for IFN-γ, compared to MS patients, and for IL-6 compared to SC (Fig 2 and S1 Table).

### Serum and CSF cytokine ratios

**Fig 3:**
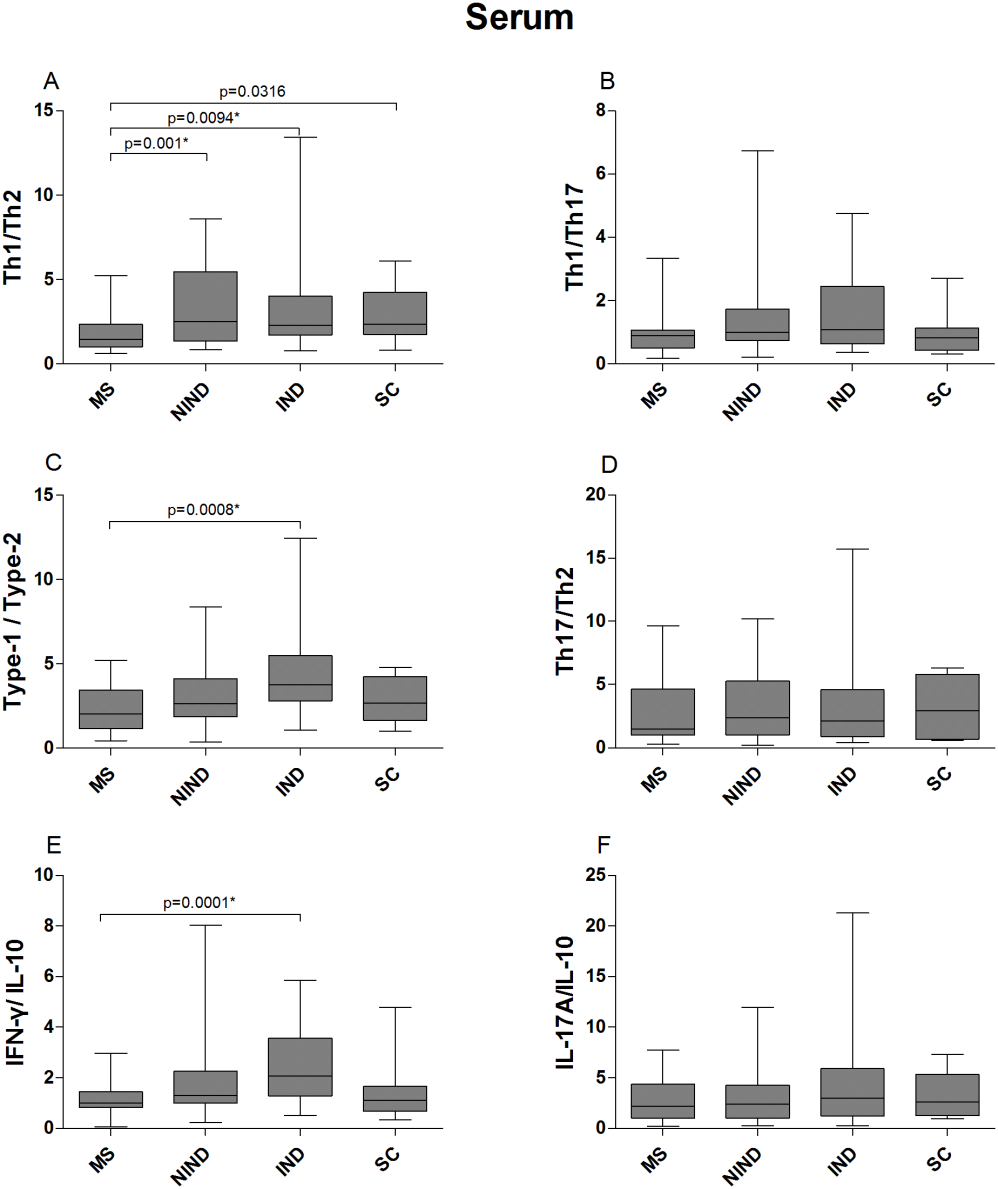
Serum cytokine ratios of MS patients and control groups. The data are presented as box plots with whiskers, showing the median with the upper (75) and lower (25) percentiles. The uncorrected p values are shown where statistical significance was reached. The asterisk (*) denotes statistical significance retained after controlling for type I error.

The serum Th1/Th2 cytokine ratio was lower in MS patients compared to the other patient groups. The differences reached statistical significance (retained after controlling for type I error) between the MS and the NIND and IND groups. The Type-1/Type-2 and IFN-γ/IL-10 ratios were significantly lower in MS patients compared to IND patients. The Th1/Th17, Th17/Th2 and IL-17/IL-10 ratios did not differ significantly between the patient groups (Fig 3 and S2 Table).

**Fig 4:**
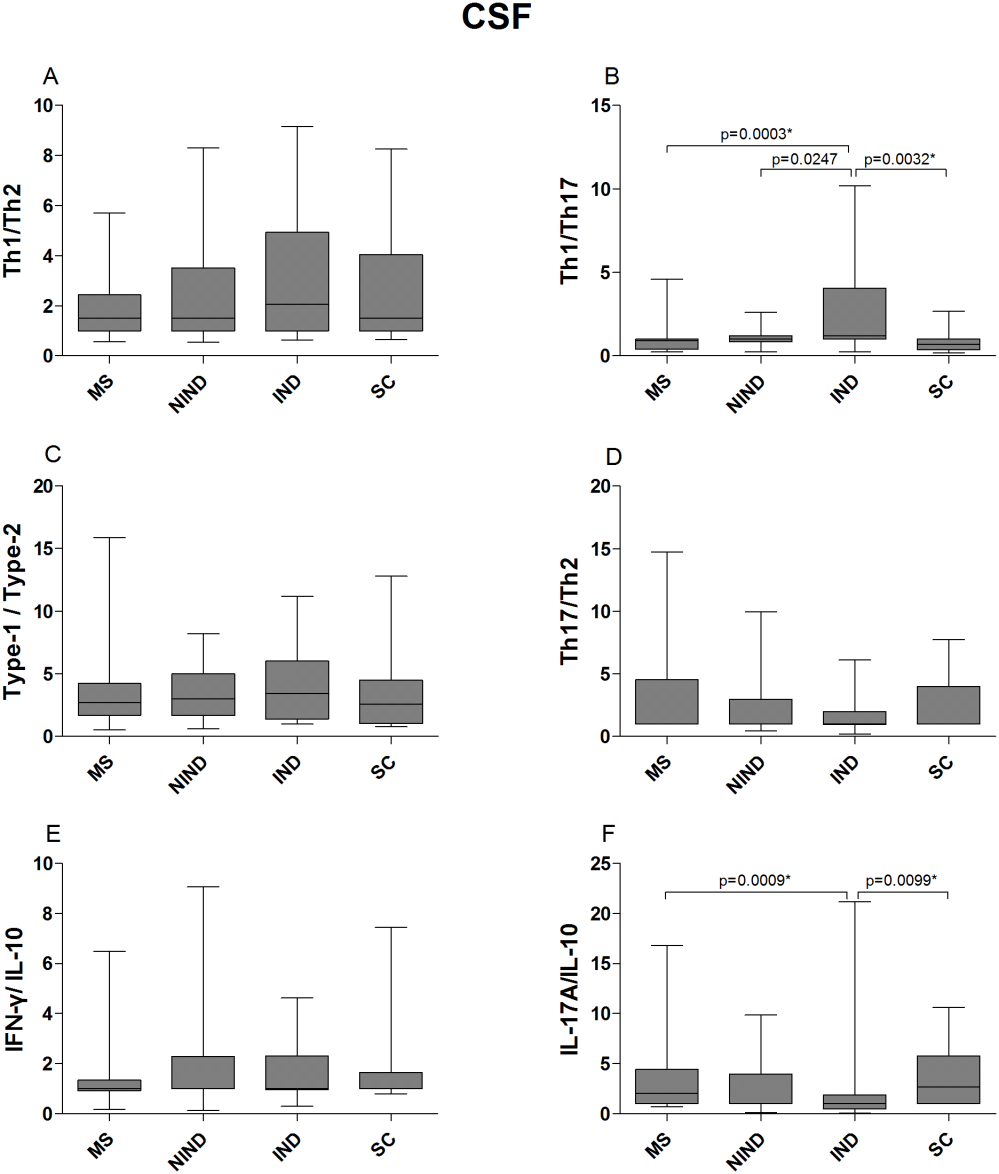
CSF cytokine ratios of MS patients and control groups. The data are presented as box plots with whiskers, showing the median with the upper (75) and lower (25) percentiles. The uncorrected p values are shown where statistical significance was reached. The asterisk (*) denotes statistical significance retained after controlling for type I error.

In CSF, the Th1/Th17 cytokine ratio was significantly lower in MS compared to IND patients, whereas the IL-17/IL-10 ratio was significantly higher in MS compared to IND patients. The Th1/Th17 ratio was significantly higher in IND patients compared to the MS and SC groups, whereas in IND patients the IL-17/IL-10 ratio was significantly lower compared to MS and SC patients. The Th1/Th2, Type-1/Type-2, Th17/Th2 and IFN-γ/IL-10 ratios did not differ significantly between the patient groups (Fig 4 and S2 Table).

### Intrathecal synthesis of IgG and correlations with cytokine levels

#### (i) IgG index and correlations with cytokine levels

According to the 2005 revised McDonald criteria, a positive CSF is determined by the presence of oligoclonal bands or by a positive IgG index [18]. The standard test performed in all patients for the detection of intrathecal IgG synthesis was the IgG index (performed in PUH). The samples were also sent to an outside laboratory (Eginition Hospital, Faculty of Medicine, National and Kapodistrian University of Athens, Athens, Greece) for the detection of oligoclonal bands in the CSF by isoelectric focusing; the results were conveyed as positive (more than 2 CSF-restricted IgG bands on isoelectric focusing) or negative (Table 1). The patients with a high IgG index tested positive for oligoclonal bands. In our study, a positive IgG index (>0.66) showed a sensitivity of 75% and a specificity of 88% for the diagnosis of MS.

**Fig 5:**
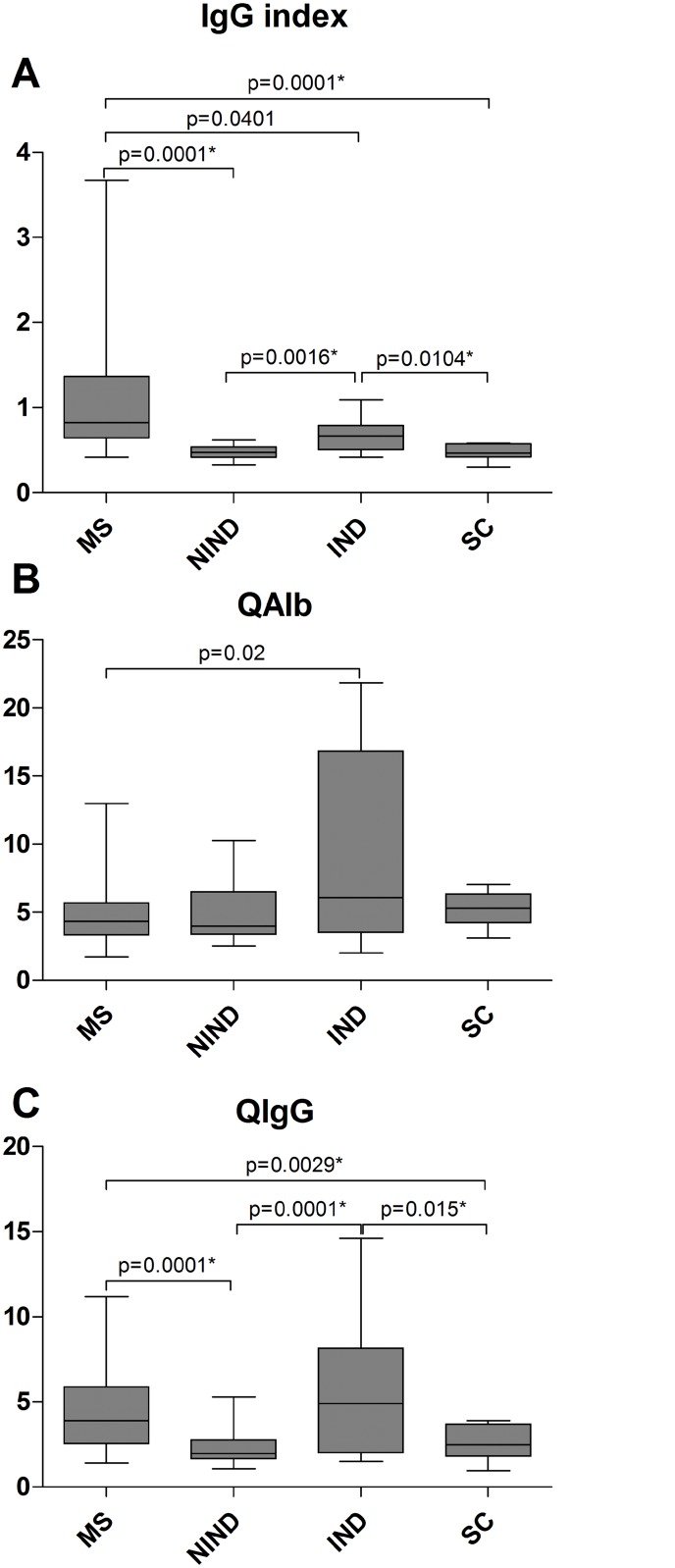
IgG index (A), QAlb (B) and QIgG (C) values of MS patients and control groups. The data are presented as box plots with whiskers, showing the median with the upper (75) and lower (25) percentiles. The uncorrected p values are shown where statistical significance was reached. The asterisk (*) denotes statistical significance retained after controlling for type I error.

**Fig 6:**
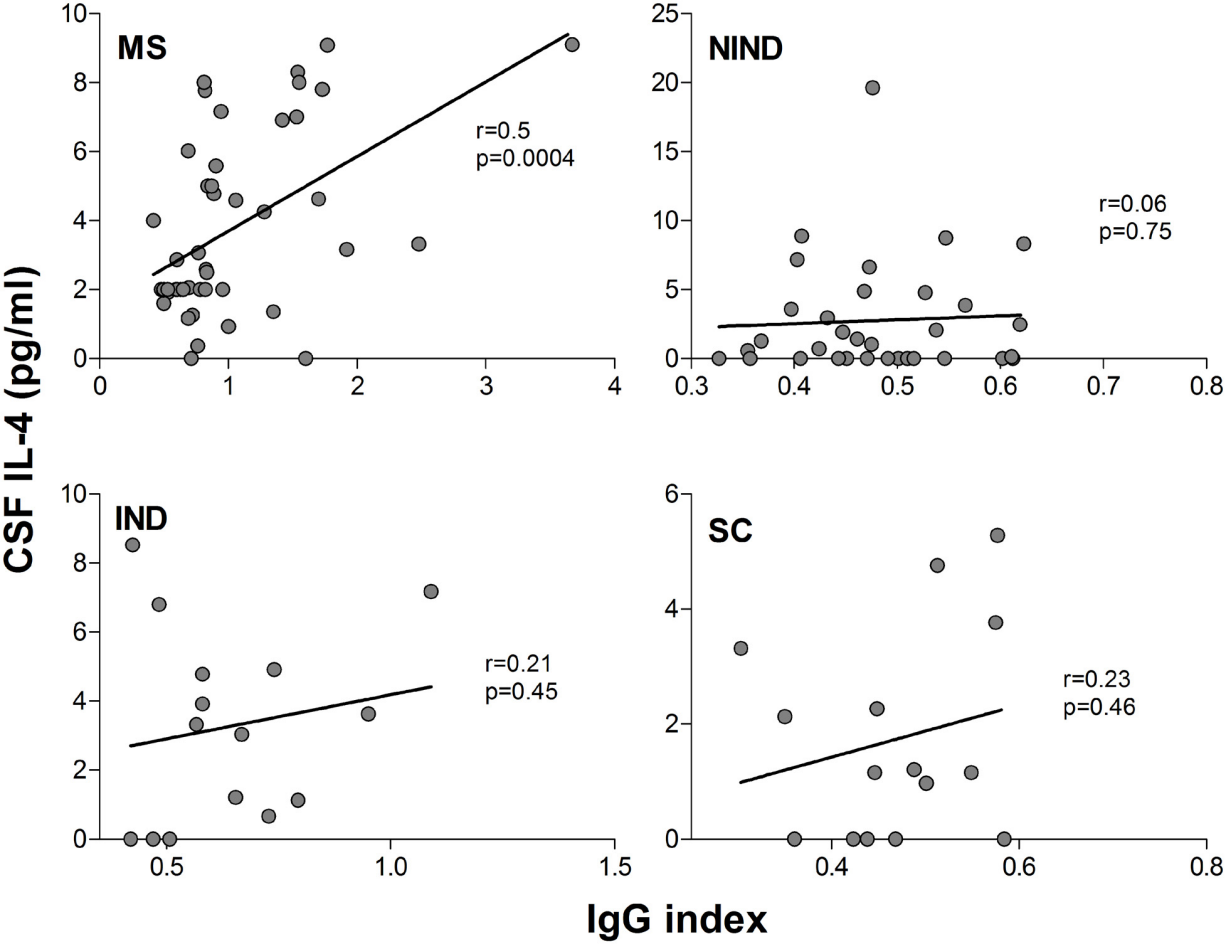
Correlation of IgG index with CSF IL-4 levels in MS patients and control groups. R-values represent Spearman’s rank correlation coefficients (with p values). The curves were fitted with linear regression using GraphPad Prism 5.03.

MS patients had the highest values for the IgG index compared to all other groups; the differences reached statistical significance (retained after controlling for type I error) between the MS and the NIND and SC groups (Fig 5A). The IgG index correlated with IL-4 levels in the CSF of MS patients only (Fig 6, r = 0.50, p = 0.0004; cf. S8 Fig for scatter plots of CSF IL-4 values of all study groups). In IND patients, the IgG index correlated with serum IL-2 (r = 0.76, p = 0.01) and CSF IFN-γ (r = 0.67, p = 0.02). There were no significant correlations between the IgG index and other serum or CSF cytokines or ratios thereof, with the exception of a negative correlation with the CSF Th1/Th17 ratio in the SC group (r = -0.69, p = 0.01) (Table 3).

**Table 3:** Correlations between IgG index and cytokines (pg/ml) or cytokine ratios in the serum and CSF of MS, NIND, IND and SC groups.

			Cytokines								Cytokine ratios					
			IFN-γ	TNF-α	IL-2	IL-6	IL-17A	IL-4	IL-10	TGF-β1	Th1/Th2	Th1/Th17	Type 1/Type 2	Th17/Th2	IFN-γ/IL-10	IL-17/IL-10
**IgG index**	**MS**	Serum	0.003 (0.98)	-0.06 (0.64)	0.30 (0.07)	0.21 (0.17)	-0.07 (0.69)	-0.07 (0.68)	-0.001 (0.99)	-0.19 (0.26)	-0.05 (0.72)	0.11 (0.46)	-0.02 (0.88)	-0.11 (0.45)	0.009 (0.95)	-0.15 (0.31)
		CSF	0.01 (0.96)	0.25 (0.23)	-0.21 (0.28)	0.08 (0.59)	0.004 (0.98)	**0.50 (0.0004)**	-0.13 (0.50)	0.02 (0.90)	0.16 (0.27)	-0.07 (0.62)	0.21 (0.17)	0.20 (0.18)	-0.04 (0.75)	0.12 (0.40)
	**NIND**	Serum	0.12 (0.54)	0.28 (0.13)	-0.19 (0.30)	0.07 (0.71)	-0.02 (0.90)	-0.22 (0.24)	-0.02 (0.91)	0.01 (0.96)	0.20 (0.28)	0.09 (0.61)	0.18 (0.33)	0.09 (0.63)	0.20 (0.28)	-0.10 (0.59)
		CSF	0.07 (0.71)	-0.07 (0.72)	0.17 (0.36)	0.06 (0.76)	-0.24 (0.20)	0.06 (0.75)	0.26 (0.17)	0.39 (0.08)	0.009 (0.96)	0.28 (0.13)	0.04 (0.81)	-0.13 (0.46)	-0.15 (0.41)	-0.06 (0.75)
	**IND**	Serum	0.17 (0.62)	0.14 (0.68)	**0.76 (0.01)**	-0.16 (0.64)	0.004 (0.99)	0.33 (0.32)	-0.29 (0.38)	0.66 (0.16)	-0.21 (0.52)	0.16 (0.61)	0.12 (0.71)	-0.25 (0.45)	-0.13 (0.69)	-0.03 (0.92)
		CSF	**0.67 (0.02)**	**0.60 (0.05)**	0.34 (0.31)	-0.15 (0.66)	0.05 (0.87)	0.21 (0.45)	0.35 (0.29)	0.20 (0.75)	0.54 (0.08)	0.52 (0.10)	-0.24 (0.46)	0.01 (0.96)	0.18 (0.59)	-0.31 (0.34)
	**SC**	Serum	-0.05 (0.89)	-0.18 (0.59)	-0.55 (0.08)	-0.30 (0.37)	-0.44 (0.17)	0.02 (0.96)	-0.53 (0.10)	0.15 (0.70)	0.10 (0.75)	0.0 (1.0)	-0.18 (0.59)	-0.34 (0.29)	0.31 (0.34)	0.02 (0.93)
		CSF	-0.04 (0.90)	0.14 (0.69)	-0.04 (0.90)	-0.31 (0.36)	0.37 (0.26)	0.23 (0.46)	-0.17 (0.61)	0.07 (0.86)	-0.08 (0.80)	**-0.69 (0.01)**	0.13 (0.68)	0.30 (0.36)	-0.36 (0.26)	0.49 (0.12)

Results are given as Spearman r values (p); numbers in bold denote statistical significance.

#### (ii) QIgG and QAlb indices and correlations with cytokine levels

**Fig 7:**
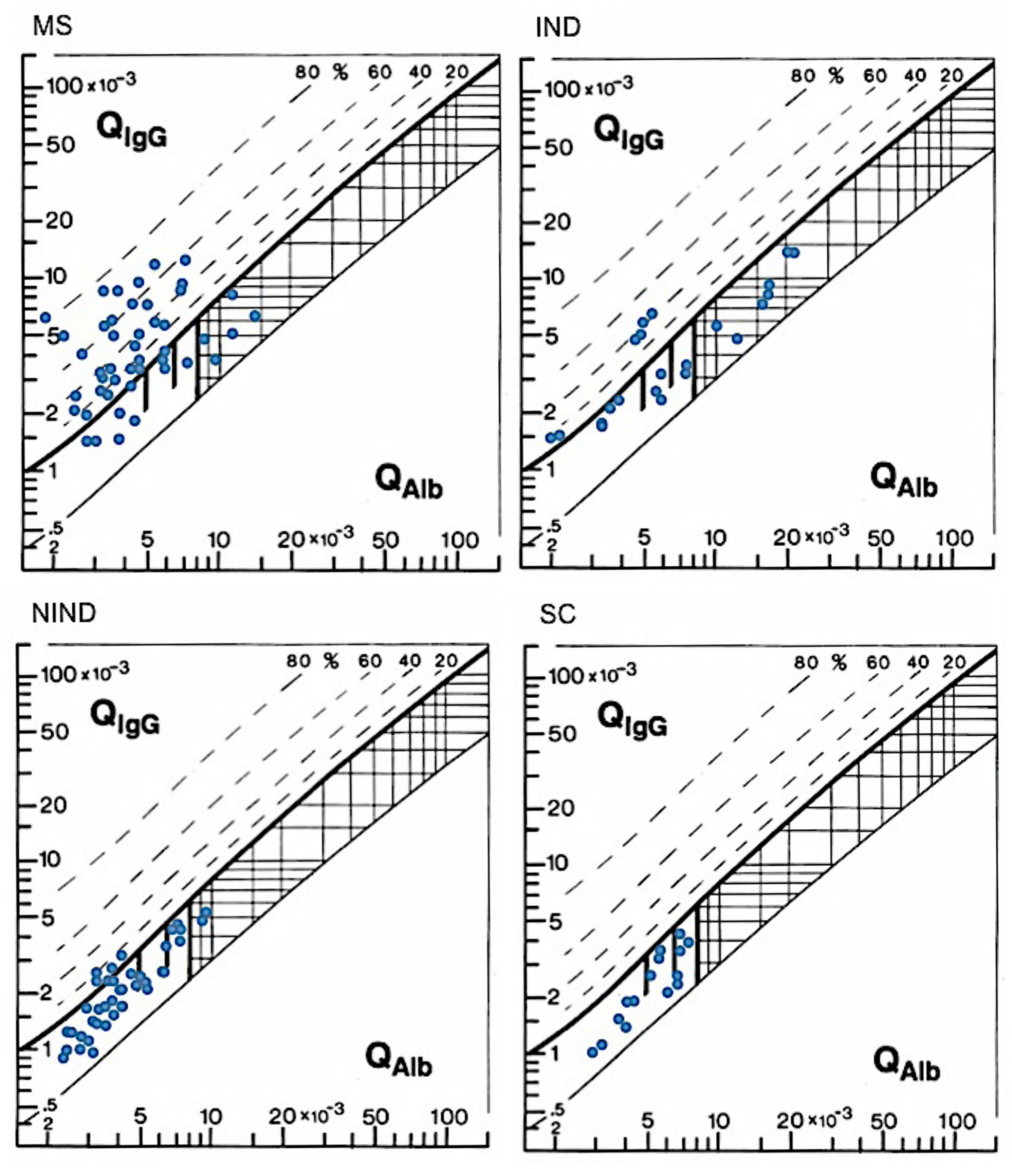
CSF/serum quotient diagrams for IgG with hyperbolic graphs (Reibergrams) for MS patients and control groups.

The disease-related IgG patterns with reference to albumin were also depicted for the 4 study groups following the method of Reiber-Felgenhauer, as analyzed in [21], where QIgG = CSF IgG/serum IgG, and QAlb = CSF Alb/serum Alb. CSF/serum quotient diagrams for IgG were plotted as Reibergrams to give an approximation of the intrathecal IgG synthesis and the permeability of the blood-CSF barrier. The resulting diagrams (Fig 7) show the highest intrathecal IgG synthesis in MS patients (67%) compared to IND patients (27%), NIND patients (10%) and SC (0%), and the highest blood-CSF barrier dysfunction in IND patients (32%) compared to MS patients (11%), NIND patients (5%) and SC (0%). To note, although 4 out of 40 NIND patients showed intrathecal IgG synthesis, OCBs were negative in these cases (Table 1).

The differences between the QAlb and QIgG values between the 4 study groups are shown in Fig 5B and 5C. IND patients had the highest values for QAlb, but the differences between the groups did not reach statistical significance (retained after controlling for type I error). MS and IND patients had the highest values for QIgG; for both groups the differences reached statistical significance when compared with the NIND and SC groups (Fig 5B and 5C).

Analysis of the QIgG and QAlb indices separately for correlations with serum or CSF cytokine levels or ratios thereof, revealed no significant correlations between the QIgG values and the serum or CSF cytokine levels in the MS, IND and SC groups. In the NIND group QIgG correlated negatively with CSF IFN-γ, TNF-α and IL-17 (Table 4). There were no significant correlations between the QIgG values and the serum or CSF cytokine ratios in the MS and IND groups. In the NIND group QIgG correlated negatively with the serum Th17/Th2 ratio and the CSF Th1/Th2, Th17/Th2, IFN-γ/IL-10 and IL17A/IL-10 ratios. In SC, QIgG correlated negatively with the CSF Th1/Th17 ratio (Table 4).

**Table 4:** Correlations between QIgG and cytokines (pg/ml) or cytokine ratios in the serum and CSF of MS, NIND, IND and SC groups.

			Cytokines								Cytokine ratios					
			IFN-γ	TNF-α	IL-2	IL-6	IL-17A	IL-4	IL-10	TGF-β1	Th1/Th2	Th1/Th17	Type 1/Type 2	Th17/Th2	IFN-γ/IL-10	IL-17/IL-10
**QIgG**	**MS**	Serum	0.10 (0.57)	0.06 (0.72)	0.18 (0.27)	0.23 (0.17)	0.10 (0.55)	0.07 (0.67)	0.14 (0.39)	-0.20 (0.31)	-0.07 (0.67)	-0.05 (0.77)	0.09 (0.59)	0.02 (0.92)	-0.06 (0.73)	0.02 (0.88)
		CSF	-0.14 (0.39)	0.04 (0.79)	0.13 (0.44)	0.13 (0.45)	-0.08 (0.64)	0.21 (0.20)	0.05 (0.78)	0.19 (0.29)	0.04 (0.83)	0.02 (0.88)	0.01 (0.93)	-0.04 (0.83)	-0.02 (0.88)	-0.05 (0.78)
	**NIND**	Serum	-0.31 (0.17)	-0.04 (0.85)	0.11 (0.63)	-0.25 (0.27)	-0.37 (0.09)	-0.26 (0.24)	0.12 (0.58)	0.46 (0.08)	-0.30 (0.18)	0.18 (0.42)	-0.33 (0.13)	**-0.49 (0.02)**	-0.20 (0.37)	**-0.51 (0.02)**
		CSF	**-0.54 (0.01)**	**-0.67 (0.0006)**	-0.16 (0.47)	-0.05 (0.84)	**-0.45 (0.04)**	-0.24 (0.29)	-0.14 (0.52)	0.44 (0.10)	**-0.57 (0.006)**	0.06 (0.79)	-0.32 (0.15)	**-0.54 (0.01)**	**-0.63 (0.002)**	**-0.59 (0.004)**
	**IND**	Serum	0.24 (0.48)	0.35 (0.30)	0 (1)	0.32 (0.34)	-0.04 (0.90)	0.24 (0.47)	0.11 (0.74)	0.40 (0.50)	0.7 (0.83)	0.35 (0.28)	0.04 (0.92)	-0.18 (0.60)	-0.20 (0.56)	-0.11 (0.76)
		CSF	-0.14 (0.69)	-0.30 (0.37)	-0.15 (0.67)	0.06 (0.85)	-0.04 (0.91)	-0.43 (0.19)	0.09 (0.78)	0.80 (0.10)	-0.41 (0.21)	-0.17 (0.62)	-0.15 (0.67)	-0.06 (0.87)	-0.16 (0.64)	-0.06 (0.87)
	**SC**	Serum	0.03 (0.95)	-0.43 (0.25)	-0.65 (0.07)	-0.37 (0.34)	-0.14 (0.71)	-0.17 (0.68)	-0.60 (0.10)	0.29 (0.50)	0.26 (0.62)	-0.17 (0.74)	0.37 (0.47)	0.09 (0.87)	0.66 (0.16)	0.37 (0.47)
		CSF	0.16 (0.68)	0.51 (0.16)	0.41 (0.27)	-0.29 (0.44)	0.44 (0.25)	0.14 (0.71)	-0.02 (0.95)	-0.21 (0.62)	0.29 (0.58)	**-0.81 (0.049)**	0.31 (0.54)	-0.03 (0.96)	-0.13 (0.80)	0.58 (0.23)

Results are given as Spearman r values (p); numbers in bold denote statistical significance.

In addition, there were no significant correlations between the QAlb values and the serum or CSF cytokine levels in the MS and IND groups. In the NIND group QAlb correlated negatively with CSF IFN-γ, and in SC QAlb correlated with CSF TNF-α (S3 Table). There were no significant correlations between the QAlb values and the serum or CSF cytokine ratios in the IND group. In the MS group QAlb correlated negatively with the CSF IL-17/IL-10 ratio. In the NIND group QAlb correlated negatively with the serum Th17/Th2 ratio and the CSF Th1/Th2, Th17/Th2 and IFN-γ/IL-10 ratios. In SC, QAlb correlated negatively with the CSF Th1/Th17 and IL-17/IL-10 ratios (S4 Table).

A comparison between plots of QIL-4/QAlb (where QIL-4 = CSF IL-4/serum IL-4) (performed to incorporate the function of the blood-CSF barrier to the cytokine results), did not reveal significant differences between the 4 groups of patients (S9 Fig).

## Discussion

Various studies have investigated cytokine levels in the CSF and/or serum of MS patients vs. control groups [22–27]. In these studies different sets of cytokines were measured in mixed MS groups (RR-MS, SP-MS, PP-MS, acute phase, remission, under treatment or not) and various control groups (specific neurological disorders, NIND, IND, healthy controls), with, inevitably, variable results. In general, most of the studies showed elevated inflammatory cytokine levels in MS patients compared to healthy controls, in serum samples mostly [22–27]. However, when MS patients were compared to other IND in general, or with groups of patients with a specific IND (transverse myelitis, neuromyelitis optica, neuro-Beçhet, etc.), IND groups showed higher levels of cytokines compared to MS [22, 28–31]. These later findings are consistent with our results, which showed that IND patients had higher levels for all measured cytokines, especially in the serum.

In our study, we measured Th-type cytokine levels in serum and CSF from samples collected from neurological patients at presentation, who, following established diagnostic procedures, were later categorized as suffering from RR-MS, NIND, IND or as symptomatic controls (SC). We also calculated various cytokine ratios, because, as shown before [32, 33], individual cytokine levels are physiologically less important than relative concentrations of antagonizing cytokines, as expressed with calculated ratios that reflect the profiles of T helper cells (Th1/Th2, Th1/Th17, Th17/Th2, IFN-γ/IL-10 and IL-17/IL-10), or an overall picture of the immune response (Type-1/Type-2) since it includes cytokines the expression of which is not restricted to specific cell populations.

The aim of using these particular groups of patients at presentation, was to examine the degree of immunological involvement at the early stages of MS.

In active MS the inflammation is acute. The inflammatory diseases of the IND group have CNS involvement, even the radiculopathies and CIDP in which inflammation spreads within the spinal subarachnoid space. Diseases included in the NIND group, such as stroke and migraine, may also have an inflammatory component; however, they are not considered as inflammatory since the primary mechanism of disease is not. In the SC group we included patients with no inflammatory, structural or other abnormality of the CNS.

To note, in the case of the patient in the IND group who had CIDP, even though CIDP is an inflammatory nerve disease, it has a radiculitis component that, although anatomically belongs to the peripheral nervous system, spreads the inflammation to the subarachnoid space, hence the findings in the CSF. In addition, MS starts in the periphery where, according to the prevailing hypothesis, the auto-reactive T-cells are activated by encountering their cognate antigens (viral? molecular mimicry with myelin components?) at the beginning of the disease, as it is the case in our patients. On the other hand, viral encephalitis, myelitis and other CNS inflammatory diseases included in the IND group have predominantly a CNS inflammation. They did not have a systematic inflammatory reaction since indices of systematic inflammation (such as elevated CBC, CRP, ESR in the blood) were in most cases negative.

We found no signature cytokines or profiles thereof in MS patients at presentation. Only IND patients presented with a Th1 cytokine polarization in serum and CSF. The parameters that distinguished MS patients from patients with other neurological disorders were the high intrathecal IgG synthesis, as depicted in the CSF/serum quotient diagrams for IgG (Reibergrams) that show disease-related IgG patterns with reference to albumin [21], the high IgG index and the correlation of the IgG index with CSF IL-4 levels.

Lucchinetti et al [34] showed the heterogeneity of active MS lesions and described 4 distinct patterns with various degrees of inflammatory infiltrates, containing CD3+ T-cells, macrophages, plasma cells, complement and immunoglobulin (Ig) deposits. T-cell and macrophage inflammation characterized both patterns I and II, but the latter was distinguished by the exclusive presence of Ig (mainly IgG) and complement deposition. Patterns III and IV also had inflammatory infiltrates but were characterized by oligodendrocyte apoptosis and death, respectively. The most frequently encountered pattern was II, indicating the important role of Ig and B-cells in the pathogenesis of MS lesions.

From the analyses we performed in the present study, we were not able to determine the source of IL-4 in the CNS of the MS patients or the control groups. Intrathecal IL-4 synthesis has been demonstrated by Ponomarev et al [35], who showed IL-4 production by purified CNS mononuclear cells, the majority of which were microglial cells. In addition, IL-4 is produced by germinal center B-cells [36], and in its presence, CD40L-stimulated B-cells undergo IgG isotype switching [37]. B-cells and the antibodies they produce, play an important role in MS pathogenesis, a notion supported by the fact that intrathecal IgG synthesis (as detected by the presence of oligoclonal bands (OCBs) or elevated intrathecal IgG synthesis in the CSF) is a consistent finding in almost all MS patients [38], and from results from clinical trials with anti-CD20 monoclonal antibodies that substantially reduced new brain MRI lesions and clinical disease relapses in RR-MS patients [39, 40].

Although the presence of OCBs in the CSF and an elevated IgG index can be detected in a wide range of neurological autoimmune, infectious and structural disorders [41], their sensitivity and specificity for MS are quite high and are used as a diagnostic tool in the initial evaluation of patients with suspected MS [18, 42]. In addition, analysis of the QIgG and QAlb indices using CSF/serum quotient diagrams for IgG (Reibergrams) that give an approximation of the intrathecal IgG synthesis and the permeability of the blood-CSF barrier [21], provide more detailed information because they differentiate between (i) normal (100% in the SC group, 85% in the NIND group, 41% in the IND group and 22% in the MS group in our study), (ii) pure blood-CSF dysfunction (32% in the IND group, 11% in the MS group and 5% in the NIND group in our study), (iii) intrathecal IgG synthesis without change in CSF turnover (67% in the MS group, 27% in the IND group and 10% in the NIND group in our study, (iv) intrathecal IgG synthesis with reduced CSF turnover (none in our study) and (v) methodological fault (none in our study).

In conclusion, no signature cytokines or profiles thereof characterize MS patients at presentation i.e. no activity of specific types of cells is evident. MS patients presented with serum or CSF cytokine levels comparable to patients with NIND and SC, and lower compared to IND patients, which presented with the highest Th1 polarization. The parameters that distinguished MS patients from patients with other neurological disorders were the intrathecal IgG synthesis, the IgG index and the correlation between the IgG index and CSF IL-4 levels.

For MS patients, there is an ongoing debate as to whether the disease is driven by inflammation or neurodegeneration [43]. This is an important question because if it is resolved, it will influence the therapeutic options of the disease. Our data obtained from serum and CSF samples collected from patients at presentation, that were eventually diagnosed with MS or other non-inflammatory or inflammatory neurological diseases or symptomatic controls, indicate that at the early stages of MS, antibody activity begins the damage to the CNS followed by the cellular immune component that contributes or even predominates in established disease. We hope that future studies will show whether application of B-cell selective depletion regimens at the early stages of MS will be proven beneficial to the patients.

## Supporting Information

S1 Fig Standard curves of recombinant IFN-γ control (standard) for serum (top) and CSF (bottom). The mean fluorescence intensity (MFI) vs the concentrations of the standard (in pg/ml) were plotted using the curve fitting software CurveExpert 1.40 to generate the standard curves from which the concentration of IFN-γ/sample/MFI value was calculated.
(TIFF)

S2 Fig Standard curves of recombinant IL-2 control (standard) for serum (top) and CSF (bottom). The mean fluorescence intensity (MFI) vs the concentrations of the standard (in pg/ml) were plotted using the curve fitting software CurveExpert 1.40 to generate the standard curves from which the concentration of IL-2/sample/MFI value was calculated.
(TIFF)

S3 Fig Standard curves of recombinant IL-4 control (standard) for serum (top) and CSF (bottom). The mean fluorescence intensity (MFI) vs the concentrations of the standard (in pg/ml) were plotted using the curve fitting software CurveExpert 1.40 to generate the standard curves from which the concentration of IL-4/sample/MFI value was calculated.
(TIFF)

S4 Fig Standard curves of recombinant IL-6 control (standard) for serum (top) and CSF (bottom). The mean fluorescence intensity (MFI) vs the concentrations of the standard (in pg/ml) were plotted using the curve fitting software CurveExpert 1.40 to generate the standard curves from which the concentration of IL-6/sample/MFI value was calculated.
(TIFF)

S5 Fig Standard curves of recombinant IL-10 control (standard) for serum (top) and CSF (bottom). The mean fluorescence intensity (MFI) vs the concentrations of the standard (in pg/ml) were plotted using the curve fitting software CurveExpert 1.40 to generate the standard curves from which the concentration of IL-10/sample/MFI value was calculated.
(TIFF)

S6 Fig Standard curves of recombinant IL-17A control (standard) for serum (top) and CSF (bottom). The mean fluorescence intensity (MFI) vs the concentrations of the standard (in pg/ml) were plotted using the curve fitting software CurveExpert 1.40 to generate the standard curves from which the concentration of IL-17A/sample/MFI value was calculated.
(TIFF)

S7 Fig Standard curves of recombinant TNF-α control (standard) for serum (top) and CSF (bottom). The mean fluorescence intensity (MFI) vs the concentrations of the standard (in pg/ml) were plotted using the curve fitting software CurveExpert 1.40 to generate the standard curves from which the concentration of TNF-α/sample/MFI value was calculated.
(TIFF)

S8 Fig CSF IL-4 (pg/ml) scatter plots for MS patients and control groups. (TIFF)

S9 Fig QIL-4/QAlb plots for MS patients and control groups. QIL-4 = CSF IL4/serum IL-4; QAlb = CSF Alb/serum Alb.
(TIFF)

S1 Table Serum and CSF cytokine measurements in MS patients and control groups. (DOCX)

S2 Table Serum and CSF cytokine ratios in MS patients and control groups. (DOCX)

S3 Table Correlations between QAlb and cytokines (pg/ml) in the serum and CSF of MS patients and control groups. (DOCX)

S4 Table Correlations between QAlb and cytokine ratios in the serum and CSF of MS patients and control groups. (DOCX)
