# Correction: Immune Parameters That Distinguish Multiple Sclerosis Patients from Patients with Other Neurological Disorders at Presentation

**DOI:** 10.1371/journal.pone.0151411

**Published:** 2016-03-10

**Authors:** Athanasia Mouzaki, Maria Rodi, Nikolaos Dimisianos, Andreas Emmanuil, Dimitra Kalavrizioti, Rosa Lagoudaki, Nikolaos C. Grigoriadis, Panagiotis Papathanasopoulos

The authors wish to clarify the methodological information for this paper. Regarding the patients included in the study, the patient samples that were from the Department of Neurology, AHEPA University Hospital (Thessaloniki, Greece) were included in the control groups. Specifically, 11 were included in the IND group, 4 in the NIND group, and 1 in the SC group.

Regarding the parameters of the IgG index that were used in the analyses included in the study, i.e., IgG index, QIgG, QAlb: Because the individual values of IgG and albumin that are required for the calculation of the parameters above were missing from the patient files, the authors measured them in all of the samples in order to acquire the complete set of data needed for their analyses. This was done as follows:

1. Albumin levels were measured in a COBAS INTEGRA 400 plus analyzer (Roche Diagnostics Ltd., Rotkreuz, Switzerland) using the Immunoturbidimetric Gen.2 method. The measurements were carried out in a fully accredited private laboratory (Microbiology Laboratory of Dr. Anna I. Davanou, MD, Patras, Greece).
2. IgG levels were measured on a BD FACSArray Bioanalyzer using the Cytometric Bead Array (CBA) Human Total IgG Flex Set (BD Biosciences, San Jose, CA, USA). The measurements were carried out in Dr. Athanasia Mouzaki’s laboratory (Laboratory of Immunohematology, Division of Hematology, Department of Internal Medicine, Medical School, University of Patras).
