# Supplementary figures and images for: Immune Parameters That Distinguish Multiple Sclerosis Patients from Patients with Other Neurological Disorders at Presentation
Source: PLoS One. 2015 Aug 28;10(8):e0135434. doi: 10.1371/journal.pone.0135434 (PMC4552669; doi:10.1371/journal.pone.0135434)

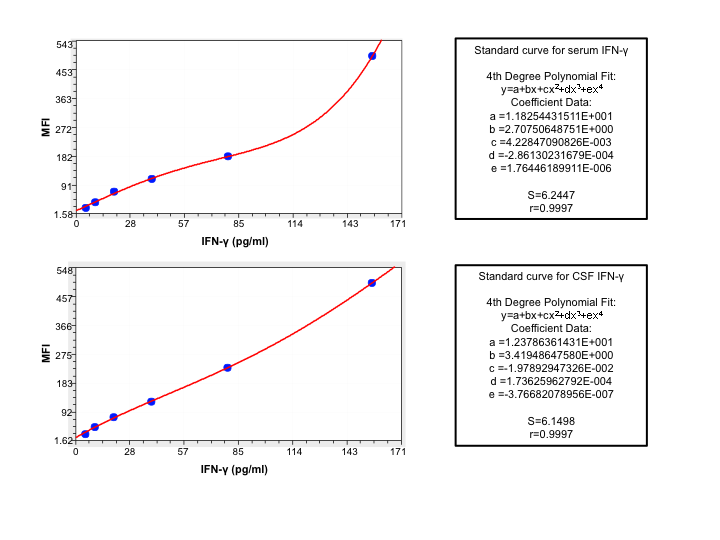

Supplement: S1 Fig — The mean fluorescence intensity (MFI) vs the concentrations of the standard (in pg/ml) were plotted using the curve fitting software CurveExpert 1.40 to generate the standard curves from which the concentration of IFN-γ/sample/MFI value was calculated. (TIFF) [file pone.0135434.s001.tiff]

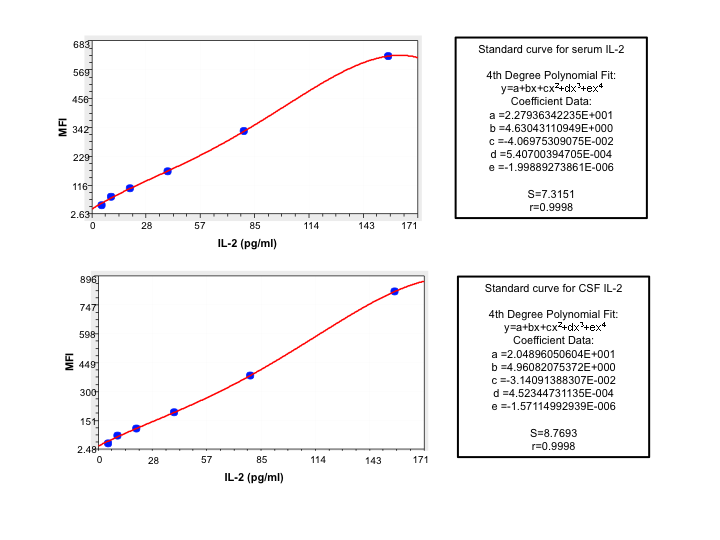

Supplement: S2 Fig — The mean fluorescence intensity (MFI) vs the concentrations of the standard (in pg/ml) were plotted using the curve fitting software CurveExpert 1.40 to generate the standard curves from which the concentration of IL-2/sample/MFI value was calculated. (TIFF) [file pone.0135434.s002.tiff]

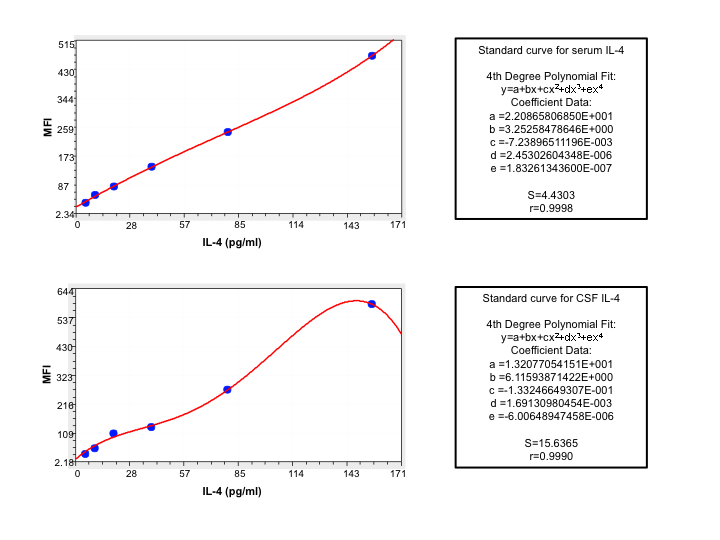

Supplement: S3 Fig — The mean fluorescence intensity (MFI) vs the concentrations of the standard (in pg/ml) were plotted using the curve fitting software CurveExpert 1.40 to generate the standard curves from which the concentration of IL-4/sample/MFI value was calculated. (TIFF) [file pone.0135434.s003.tiff]

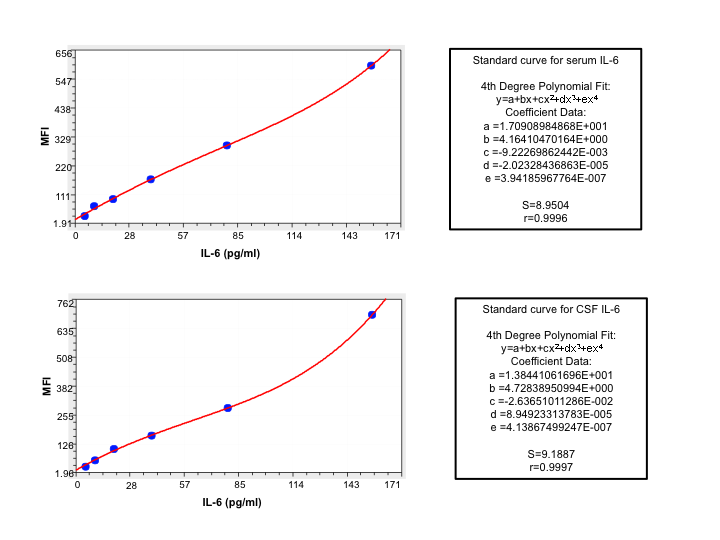

Supplement: S4 Fig — The mean fluorescence intensity (MFI) vs the concentrations of the standard (in pg/ml) were plotted using the curve fitting software CurveExpert 1.40 to generate the standard curves from which the concentration of IL-6/sample/MFI value was calculated. (TIFF) [file pone.0135434.s004.tiff]

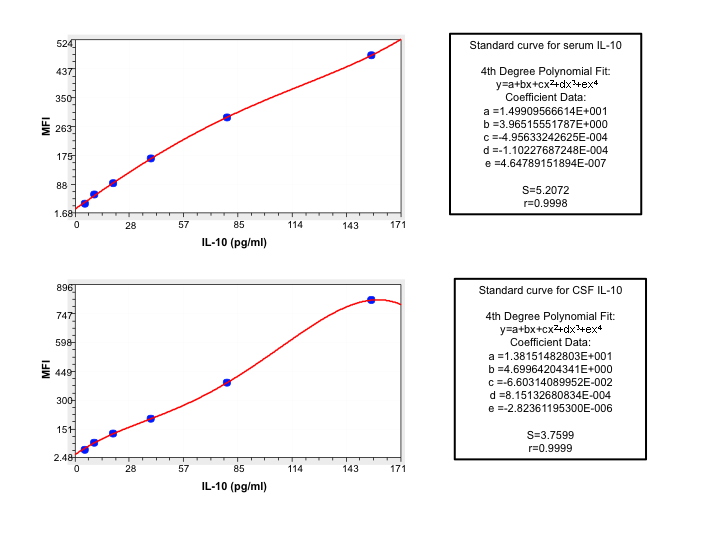

Supplement: S5 Fig — The mean fluorescence intensity (MFI) vs the concentrations of the standard (in pg/ml) were plotted using the curve fitting software CurveExpert 1.40 to generate the standard curves from which the concentration of IL-10/sample/MFI value was calculated. (TIFF) [file pone.0135434.s005.tiff]

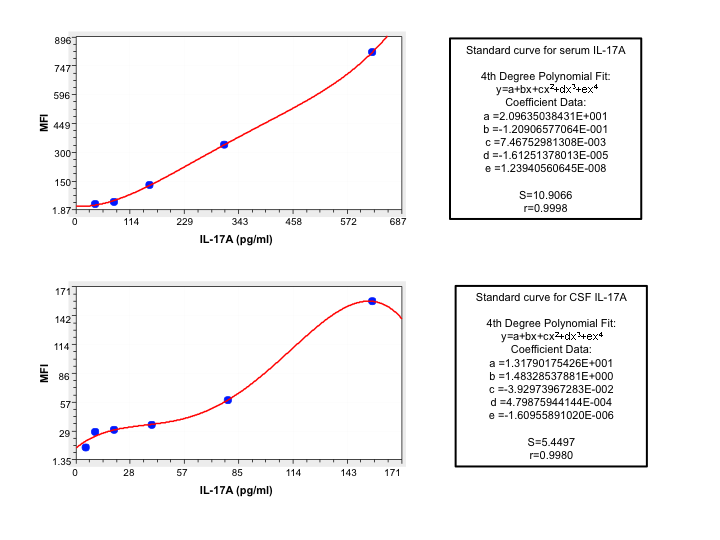

Supplement: S6 Fig — The mean fluorescence intensity (MFI) vs the concentrations of the standard (in pg/ml) were plotted using the curve fitting software CurveExpert 1.40 to generate the standard curves from which the concentration of IL-17A/sample/MFI value was calculated. (TIFF) [file pone.0135434.s006.tiff]

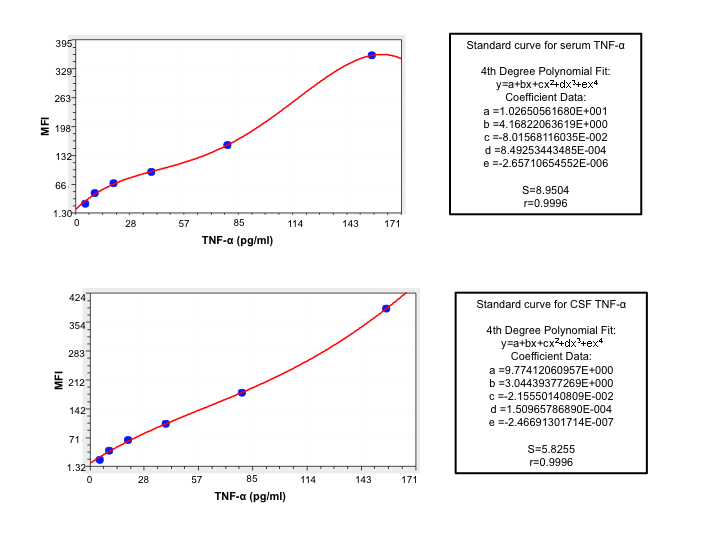

Supplement: S7 Fig — The mean fluorescence intensity (MFI) vs the concentrations of the standard (in pg/ml) were plotted using the curve fitting software CurveExpert 1.40 to generate the standard curves from which the concentration of TNF-α/sample/MFI value was calculated. (TIFF) [file pone.0135434.s007.tiff]
