# Supplementary material for: Immune Parameters That Distinguish Multiple Sclerosis Patients from Patients with Other Neurological Disorders at Presentation
Source: PLoS One. 2015 Aug 28;10(8):e0135434. doi: 10.1371/journal.pone.0135434 (PMC4552669; doi:10.1371/journal.pone.0135434)
Supplement: S1 Table — (DOCX) [file pone.0135434.s010.docx]

**Table S1.** Serum and CSF cytokine measurements in MS patients and control groups

| Group | Serum Cytokines | | | | | | | |
| --- | --- | --- | --- | --- | --- | --- | --- | --- |
|  | **IFNγ** | **TNFα** | **IL-2** | **IL-6** | **IL-17A** | **IL-4** | **IL-10** | **TGFβ1** |
| MS (n=46) | 6.02 ± 4.07 | 5.42 ± 4.07 | 4.93 ± 2.60 | 7.72 ± 4.59 | 17.07 ± 11.13 | 4.67 ± 2.35 | 6.03 ± 7.64 | 38962 ± 11082 |
| NIND (n=40) | 8.45±6.38 | 8.31± 7.95 | 4.87 ± 6.39 | 7.34 ± 3.90 | 13.23 ±12.30 | 5.23 ± 2.78 | 4.99 ± 5.94 | 32359 ± 6005 |
| IND (n=22) | 20.32±24.74 | 15.44 ± 20.29 | 17.75 ± 17.62 | 13.60 ± 11.68 | 39.25 ± 67.03 | 12.65 ± 12.90 | 13.30 ± 34.85 | 41006 ± 13358 |
| SC (n=15) | 7.05±5.87 | 6.86 ± 5.15 | 6.41 ± 5.70 | 7.71 ± 4.38 | 15.86 ± 17.35 | 5.27 ± 2.56 | 5.81 ± 4.28 | 34499 ± 10041 |
|  | **CSF Cytokines** | | | | | | | |
| MS (n=46) | 5.67 ± 6.19 | 5.34 ± 4.13 | 2.46 ± 2.46 | 6.04 ± 4.86 | 16.53 ± 16.72 | 3.30 ± 2.38 | 4.34 ± 4.99 | 74.59 ± 33.09 |
| NIND (n=40) | 6.33 ± 7.23 | 3.38±4.26 | 4.04 ± 7.90 | 11.66±12.24 | 8.52 ±11.99 | 3.83±5.29 | 4.20±11.43 | 94.25 ± 38.15 |
| IND (n=22) | 13.06 ± 17.36 | 7.56 ± 7.70 | 11.31 ± 10.39 | 18.31 ± 24.81 | 8.69 ± 11.60 | 6.51±7.09 | 14.80±24.25 | 215.4±252 |
| SC (n=15) | 3.72 ± 6.90 | 1.95± 3.21 | 4.97 ± 4.00 | 6.24 ± 5.08 | 13.69 ± 26.23 | 1.74 ± 1.93 | 0.38±1.06 | 64.06 ± 37.07 |

Data are given as mean±SD in pg/ml
