# Supplementary material for: Immune Parameters That Distinguish Multiple Sclerosis Patients from Patients with Other Neurological Disorders at Presentation
Source: PLoS One. 2015 Aug 28;10(8):e0135434. doi: 10.1371/journal.pone.0135434 (PMC4552669; doi:10.1371/journal.pone.0135434)
Supplement: S2 Table — (DOCX) [file pone.0135434.s011.docx]

**Table S2.** Serum and CSF cytokine ratios in MS patients and control groups

| Group | | Serum ratios | | | | | | | | | | | |
| --- | --- | --- | --- | --- | --- | --- | --- | --- | --- | --- | --- | --- | --- |
|  |  | **Th1/Th2** | | **Th1/Th17** | | **Type 1/Type 2** | | **Th17/Th2** | | **IFNγ/IL-10** | | **IL-17A/IL-10** | |
| MS (n=46) | | 2.05 ± 1.19 | | 0.90 ± 0.64 | | 2.36 ± 1.35 | | 2.81 ± 2.33 | | 1.28 ± 0.70 | | 3.18 ± 2.05 | |
| NIND (n=40) | | 3.41 ± 2.35 | | 1.43 ± 1.26 | | 3.07 ± 1.86 | | 3.22 ± 2.65 | | 2.06 ± 1.83 | | 3.21 ± 2.82 | |
| IND (n=22) | | 3.31 ± 2.85 | | 1.62 ± 1.24 | | 4.21 ± 2.49 | | 3.17 ± 3.45 | | 2.47 ± 1.49 | | 4.14 ± 4.83 | |
| SC (n=15) | | 2.85 ± 1.62 | | 0.96 ± 0.68 | | 2.89 ± 1.32 | | 3.13 ± 2.45 | | 1.46 ± 1.26 | | 3.16 ± 2.28 | |
|  | **CSF ratios** | | | | | | | | | | | |  |
| MS (n=46) | 2.50 ± 1.29 | | 0.88 ± 0.89 | | 3.30 ±2.48 | | 2.54 ± 2.18 | | 1.42 ± 1.21 | | 4.85 ±4.07 | |  |
| NIND (n=40) | 2.48 ± 2.00 | | 1.08 ± 0.49 | | 3.34 ± 2.06 | | 2.05± 1.97 | | 1.82 ± 1.58 | | 2.70 ± 2.67 | |  |
| IND (n=22) | 3.04 ± 2.50 | | 2.72 ± 2.94 | | 3.85 ± 2.69 | | 1.79 ± 1.55 | | 1.51 ± 1.15 | | 2.34 ± 4.53 | |  |
| SC (n=15) | 2.61 ± 2.39 | | 0.81 ± 0.66 | | 3.75 ± 3.43 | | 2.38 ± 2.20 | | 1.82 ± 1.91 | | 3.73 ± 3.41 | |  |

Values are given as mean±SD
