# Supplementary material for: Immune Parameters That Distinguish Multiple Sclerosis Patients from Patients with Other Neurological Disorders at Presentation
Source: PLoS One. 2015 Aug 28;10(8):e0135434. doi: 10.1371/journal.pone.0135434 (PMC4552669; doi:10.1371/journal.pone.0135434)
Supplement: S3 Table — (DOCX) [file pone.0135434.s012.docx]

|  |  |  | **Cytokines** | | | | | | | |
| --- | --- | --- | --- | --- | --- | --- | --- | --- | --- | --- |
|  |  |  | **IFN-γ** | **TNF-α** | **IL-2** | **IL-6** | **IL-17A** | **IL-4** | **IL-10** | **TGF-β1** |
| **QIgG** | **MS** | Serum | 0.07 (0.66) | 0.16 (0.34) | 0.07 (0.66) | 0.06 (0.74) | 0.17 (0.31) | 0.05 (0.77) | 0.01 (0.94) | -0.05 (0.81) |
|  |  | CSF | -0.12 (0.46) | -0.24 (0.14) | -0.06 (0.71) | 0.08 (0.62) | -0.32 (0.06) | -0.08 (0.65) | -0.14 (0.39) | 0.24 (0.18) |
|  | **NIND** | Serum | -0.09 (0.64) | -0.10 (0.61) | 0.30 (0.12) | -0.08 (0.68) | -0.24 (0.21) | -0.002 (0.99) | 0.03 (0.88) | 0.31 (0.24) |
|  |  | CSF | **-0.41 (0.03)** | -0.36 (0.06) | 0.16 (0.42) | 0.18 (0.35) | -0.14 (0.49) | 0.09 (0.66) | -0.16 (0.42) | 0.41 (0.12) |
|  | **IND** | Serum | 0.22 (0.38) | 0.15 (0.53) | -0.22 (0.36) | 0.25 (0.30) | -0.23 (0.34) | -0.10 (0.68) | 0.09 (0.73) | 0.40 (0.50) |
|  |  | CSF | 0.15 (0.55) | -0.06 (0.81) | -0.04 (0.87) | 0.29 (0.23) | 0.09 (0.71) | -0.02 (0.94) | 0.04 (0.86) | 0.80 (0.10) |
|  | **SC** | Serum | 0.18 (0.73) | -0.27 (0.49) | -0.51 (0.16) | -0.23 (0.55) | 0.09 (0.82) | -0.03 (0.95) | -0.47 (0.21) | 0.12 (0.78) |
|  |  | CSF | 0.39 (0.30) | **0.69 (0.04)** | 0.41 (0.27) | -0.43 (0.25) | 0.57 (0.11) | 0.07 (0.66) | -0.02 (0.95) | -0.31 (0.46) |

**Table S3.** Correlations between QAlb and cytokines (pg/ml) in the serum and CSF of MS patients and control groups
Results are given as Spearman r values (p); numbers in bold denote statistical significance.
