# Supplementary material for: Immune Parameters That Distinguish Multiple Sclerosis Patients from Patients with Other Neurological Disorders at Presentation
Source: PLoS One. 2015 Aug 28;10(8):e0135434. doi: 10.1371/journal.pone.0135434 (PMC4552669; doi:10.1371/journal.pone.0135434)
Supplement: S4 Table — (DOCX) [file pone.0135434.s013.docx]

**Table S4.** Correlations between QAlb and cytokine ratios in the serum and CSF of MS patients and control groups

|  |  |  | **Cytokine ratios** | | | | | |
| --- | --- | --- | --- | --- | --- | --- | --- | --- |
|  |  |  | **Th1/Th2** | **Th1/Th17** | **Type 1/Type 2** | **Th17/Th2** | **IFNγ/IL-10** | **IL-17A/IL-10** |
| **QIgG** | **MS** | Serum | 0.002 (0.99) | -0.02 (0.90) | 0.09 (0.59) | 0.08 (0.65) | -0.10 (0.56) | 0.05 (0.76) |
|  |  | CSF | -0.19 (0.24) | 0.14 (0.42) | -0.22 (0.18) | -0.26 (0.11) | -0.09 (0.60) | **-0.36 (0.03)** |
|  | **NIND** | Serum | -0.27 (0.16) | 0.15 (0.45) | -0.21 (0.29) | **-0.42 (0.03)** | -0.05 (0.81) | -0.30 (0.12) |
|  |  | CSF | **-0.54 (0.003)** | 0.02 (0.93) | -0.25 (0.20) | **-0.38 (0.047)** | **-0.46 (0.01)** | -0.36 (0.06) |
|  | **IND** | Serum | 0.20 (0.42) | -0.12 (0.63) | 0.08 (0.76) | -0.18 (0.60) | 0.02 (0.94) | -0.24 (0.32) |
|  |  | CSF | -0.13 (0.58) | -0.07 (0.77) | 0.01 (0.98) | -0.06 (0.87) | -0.06 (0.82) | 0.01 (0.96) |
|  | **SC** | Serum | 0.12 (0.76) | -0.19 (0.61) | 0.33 (0.39) | 0.09 (0.81) | 0.65 (0.07) | 0.45 (0.23) |
|  |  | CSF | 0.48 (0.19) | **-0.73 (0.03)** | 0.63 (0.08) | 0.37 (0.33) | -0.18 (0.64) | **0.72 (0.03)** |

Results are given as Spearman r values (p); numbers in bold denote statistical significance
